# Wide variation in the suboptimal distribution of photosynthetic capacity in relation to light across genotypes of wheat

**DOI:** 10.1093/aobpla/plaa039

**Published:** 2020-08-11

**Authors:** William T Salter, Andrew Merchant, Richard M Trethowan, Richard A Richards, Thomas N Buckley

**Affiliations:** 1 School of Life and Environmental Sciences, Sydney Institute of Agriculture, The University of Sydney, Brownlow Hill, NSW, Australia; 2 CSIRO Agriculture and Food, Canberra, ACT, Australia; 3 Department of Plant Sciences, University of California, Davis, Davis, CA, USA

**Keywords:** Breeding, canopy, nitrogen, optimality, photosynthesis, wheat

## Abstract

Suboptimal distribution of photosynthetic capacity in relation to light among leaves reduces potential whole-canopy photosynthesis. We quantified the degree of suboptimality in 160 genotypes of wheat by directly measuring photosynthetic capacity and daily irradiance in flag and penultimate leaves. Capacity per unit daily irradiance was systematically lower in flag than penultimate leaves in most genotypes, but the ratio (*γ*) of capacity per unit irradiance between flag and penultimate leaves varied widely across genotypes, from less than 0.5 to over 1.2. Variation in *γ* was most strongly associated with differences in photosynthetic capacity in penultimate leaves, rather than with flag leaf photosynthesis or canopy light penetration. Preliminary genome-wide association analysis identified nine strong marker-trait associations with this trait, which should be validated in future work in other environments and/or materials. Our modelling suggests canopy photosynthesis could be increased by up to 5 % under sunny conditions by harnessing this variation through selective breeding for increased *γ*.

## Teaser

Plants invest nitrogen in photosynthetic enzymes in leaves. Although the optimal investment should be roughly proportional to how much light each leaf receives, plants tend to underinvest N in upper-canopy (sunlit) leaves. We examined, for the first time, whether this suboptimal pattern differs within species, by measuring light capture and photosynthetic capacity in flag and penultimate leaves across 160 wheat genotypes. We found wide variation in suboptimal N distribution, mostly driven by variation in plants’ ability to move N from penultimate to flag leaves as flag leaves develop. Selecting for less-suboptimal N distribution could increase canopy photosynthesis by several percent.

## Introduction

Food production must increase greatly in coming decades to feed a growing population (Fischer and Edmeades 2010; Ray *et al.* 2012, 2013; Long *et al.* 2015). Crop growth and yield are ultimately limited by carbohydrate supply from canopy photosynthesis, which in turn is limited by photosynthetic inputs, chiefly water, light and nitrogen, which is needed to build photosynthetic enzymes. Efforts to improve crop photosynthesis often focus on increasing leaf photosynthesis rate per unit input (Furbank *et al.* 2015), for example by enhancing Rubisco function (Parry *et al.* 2003; Whitney *et al.* 2010), accelerating recovery from energy-consuming photoprotection (Zhu *et al.* 2004; Murchie and Niyogi 2010; Kromdijk *et al.* 2016) or enhancing CO_2_ transport to the sites of carboxylation (Flexas *et al.* 2013; Jahan *et al.* 2014; McGrath and Long 2014). Yet crop photosynthesis depends not only on the photosynthetic rate per unit input, but also on the contributions of leaves with widely varying inputs, in terms of light (Goudriaan 1977) and photosynthetic N. For a given total N, whole-canopy photosynthesis is maximized if N is distributed such that the marginal revenue of N (∂*A*_day_/∂N, where *A*_day_ is leaf daily net photosynthesis) is invariant among leaves (Field 1983). This is approximately equivalent to invariance in the ratio of photosynthetic capacity (*A*_m_, light- and CO_2_-saturated leaf net photosynthesis rate) to daily irradiance (*i*_d_, daily absorbed photosynthetic photon flux), or capacity per unit irradiance (*A*_m_/*i*_d_) (Farquhar 1989; Anten *et al.* 1995; Badeck 1995; Sands 1995). Real canopies in nature diverge systematically from this optimum, capacity per unit irradiance being greater in more shaded positions within a canopy and smaller in more sunlit positions (Niinemets 2007, 2012), in both woody (e.g. Hollinger 1996; Bond and Kavanagh 1999; Friend 2001; Frak *et al.* 2002; Lloyd *et al.* 2010) and herbaceous species (e.g. Hirose and Werger 1987, 1994; de Pury and Farquhar 1997; Makino *et al.* 1997).

The mechanistic basis of suboptimal canopy *A*_m_ distribution and its implications for fitness have long been debated, with no clear resolution. Hypotheses include systematic depression of stomatal conductance in upper canopy locations by water transport limitations (Peltoniemi *et al.* 2012; Buckley *et al.* 2013); limited capacity to retranslocate N as leaves senesce and/or become shaded by other leaves (Niinemets 2007); limits on the magnitude of *A*_m_ in sunlit locations (Lloyd *et al.* 2010), perhaps arising from limits on leaf mass per unit area (Dewar *et al.* 2012); limits on the capacity to optimally balance N-requiring components of photosynthesis such as light harvesting, carboxylation and electron transport (Kull 2002); and costs or benefits that are typically excluded from models, such as the metabolic costs of retranslocation (e.g. Kull and Kruijt 1998; Dewar *et al.* 2012), the benefit of leaf area proliferation for light competition (Schieving and Poorter 1999; Anten 2002), the N cost of light harvesting (Badeck 1995; Buckley and Farquhar 2004) or the effect of leaf N on herbivory risk (Stockhoff 1994). It is unclear whether any one of these hypotheses can explain the divergence of capacity profiles from calculated optima in all vegetation types and ecological contexts (Niinemets 2007, 2012).

If calculated optima are approximately correct, and thus capacity distributions are indeed systematically suboptimal, this may represent an opportunity for crop improvement. Simulations suggest that canopy carbon gain could increase without additional N inputs if canopy *A*_m_ profiles were adjusted to match theoretical optima (de Pury and Farquhar 1997; Buckley *et al.* 2013; Townsend *et al.* 2018). Yet, for the genetic resources available to breeders, little is known about heritable variability among genotypes in the degree to which capacity profiles are optimal. No study, to our knowledge, has examined this question in more than two genotypes of the same species (Townsend *et al.* 2018).

Here we report the first assessment of genetically linked variation in this trait across diverse germplasm. We measured *A*_m_ and *i*_d_ in flag leaves and penultimate leaves (the leaf rank immediately below flag leaves) in 160 genotypes of wheat grown under field conditions in eastern Australia, and used a genome-wide association study to identify preliminary genetic markers linked to variation in the ratio of capacity per unit irradiance between flag and penultimate leaves (*γ*). Our data—the first survey of within-canopy variation in capacity per unit irradiance across diverse genotypes, and also the widest direct survey to date of photosynthetic capacity across genotypes of any given species grown under field conditions—revealed more than 2.5-fold variation in *γ* across genotypes, and identified seven chromosome locations potentially linked to this variation.

## Materials and Methods

### Plant material

Wheat was planted in 2 × 6 m plots with five sowing rows per plot. Two weeks before measurements began, access lanes were mowed between ranges of plots, leaving each plot 2 × 4 m in size for measurement and later harvest. Two to three buffer rows and ranges were planted at the outer margins of the planting area. Two hundred fifty genotypes were planted, with two plots per genotype. Two hundred fifty plots (one per genotype) were planted in a block of 17 rows × 16 ranges, including one range of buffer. Another 250 plots (a second replicate for each genotype) were planted in an adjacent block immediately south-east. **Supporting Information—Figure S1** illustrates the plot layout. Genotypes were randomly distributed within each block. Phenological development was unusually quick due to dry and warm conditions, so, to limit the phenological range of our sample, we phenotyped only 160 genotypes. These genotypes were selected based on the need to phenotype canopy light environment (as described below) for both replicate plots for each genotype within a period of 12 days. Because the logistics of phenotyping the light environment required that we work on two adjacent ranges of 17 rows each day, we were restricted to measuring genotypes that occurred twice (two replicate plots) within a finite set of 12 complete ranges. Thus, the set of genotypes on which we measured both photosynthetic capacity and light environment was, in effect, a consequence of the random distribution of genotypes within each block. The distribution of phenological stages across the measurement campaign is shown in **Supporting Information—Fig. S2**; the median Zadok growth stages were 59 (ear emergence complete) and 65 (anthesis half-way) for the first and second blocks of replicate plots for each genotype, measured on 3–10 and 11–18 September 2017, respectively.

The 160 genotypes studied in this work arose from three sources: seven Australian commercial check cultivars, 119 lines from a population created at the University of Sydney and 34 lines from a MAGIC (Multi-parent Advanced Generation Inter-cross) population created by CSIRO. The 119 Sydney lines were selected from a larger population that included 160 genotypes of *Triticum dicoccum*, 100 primary synthetic wheats with their original genome donors, synthetic-derived materials developed from crosses of primary synthetics with Indian and Australian cultivars and over 1000 fixed hexaploid progeny of *T. dicoccum* crossed with hexaploid wheat. Many of the derived genotypes are high yielding, semi-dwarf varieties. We included 34 lines from the CSIRO four-way MAGIC population. This population was developed from four Australian commercial parents (Baxter, Chara, Westonia and Yitpi), each having a low co-ancestry, by intercrossing the parents to maximize genetic diversity and recombination. Each single seed was then selfed to produce pure lines (Huang *et al.* 2012). These 34 lines were drawn from a much larger population (nearly 1600 lines) based on variation in canopy architecture, after culling to eliminate extremes in flowering time and height. We also included seven Australian commercial wheat cultivars that differed in canopy architecture. All genotypes are listed in **Supporting Information—Table S1**.

DNA of a subset of 118 of the 119 Sydney genotypes was extracted following the CTAB described by Doyle and Doyle (1990). The materials were subsequently genotyped using the Infinium iSelect SNP 90K SNP Assay (Cavanagh *et al.* 2013; Wang *et al.* 2014). The lines from the MAGIC population could not be genotyped due to proprietary commercial intellectual property concerns.

### Measurements of photosynthetic capacity (*A*_m_, CO_2_- and light-saturated net assimilation rate)

We measured photosynthetic capacity, defined here as the net rate of CO_2_ assimilation under saturating light and CO_2_ concentration and denoted *A*_m_. It is important to distinguish *A*_m_ from *V*_cmax_ and *J*_max_ (the maximum velocity of RuBP carboxylation and maximum potential electron transport rate, respectively). We chose to measure *A*_m_ rather than *V*_cmax_ or *J*_max_ because the latter require response curves of *A* vs. intercellular CO_2_ concentration (*c*_i_) and *A* vs. photosynthetic photon flux density (PPFD or *i*), which are very time-consuming and would have reduced throughput by nearly an order of magnitude, making this study impossible with the resources available to use. In this context it is worth noting that, although *V*_cmax_ and *J*_max_ provide more information than *A*_m_, *A*_m_ does have some advantages: because *V*_cmax_ and *J*_max_ are inferred from models fitted to *A* vs. *c*_i_ and *A* vs. *i* curves, inferences of *V*_cmax_ and *J*_max_ are laden with assumptions of those models, and also assumptions used to infer *c*_i_ itself from gas exchange measurements. By contrast, *A*_m_ is a direct measurement of actual photosynthetic potential.

We measured *A*_m_ using OCTOflux, an open-flow single-pass differential gas exchange system with eight leaf chambers (5 × 11 cm). This system is described elsewhere (Salter *et al.* 2018a) and summarized briefly here. Each chamber has a white LED light source (WL-18W-O60, Super Bright LEDs, Inc., St. Louis, MO, USA) positioned above the adaxial surface of the leaf, four small mixing fans (UB3F3-500, SUNON, Kaohsiung City, Taiwan), a Propafilm (#250-01885, LI-COR, Lincoln, NE, USA) window and a type T thermocouple (TT-T-36-100, Omega Engineering, Inc., Norwalk, CT, USA) appressed to the abaxial leaf surface. Compressed CO_2_ is injected into a stream of compressed dry air using a mass flow controller (MFC, FMA5412, Omega Engineering, Inc.) and mixed in a large buffering volume containing a powerful fan (PF40281B1-000U-G99, Sunon, Brea, CA, USA), before splitting into eight sample streams and a reference stream. Each sample stream runs through a mass flow meter (MFM; 822-13-0D1-PV1-V1 MFM, Sierra Instruments) to a leaf chamber and back, and then solenoid valves are used to either vent the stream to the atmosphere or direct it through the sample cell of a differential infrared gas analyzer (IRGA, Li-7000, LI-COR). One half of the reference gas stream runs through the IRGA reference cell; the other half is either vented to the atmosphere or directed through the sample cell to match the IRGA. The system is interrogated and controlled with a Microsoft Excel program that communicates with instruments via VB.NET interface functions that communicate with USB DAQ boards (USB-2416-4AO and USB-ERB24, Measurement Computing Corporation, Norton, MA, USA) and via an RS-232 serial connection (to the IRGA).

We measured *A*_m_ in both the flag and penultimate leaves (the leaf rank immediately below the flag leaf) of the same tiller, for two tillers per plot and two plots per genotype. Tillers were cut in the field, immediately recut under distilled water, returned to the laboratory (about 1 km away) and kept in darkness for 0–30 min before measurement. Each leaf was enclosed in a leaf chamber and exposed to saturating PPFD (1700 μmol m^−2^ s^−1^) and CO_2_ (4800–5000 ppm), and then allowed to adjust to these conditions until net CO_2_ assimilation rate (*A*) was stable (typically 15–20 min). Chamber flow rate was 1 L min^−1^ and leaf temperature averaged 26.0 ± 1.7 °C (mean ± SD). Photosynthetic induction was assessed by continuously monitoring *A* of one leaf. Once gas exchange rates stabilized, the gas stream from each leaf chamber was sequentially passed through the IRGA sample cell for 1 min, and *A*_m_ was taken as the average of *A* over the last 40 s. One measurement cycle (eight leaves plus matching) took 28.7 ± 5.8 min (mean ± SD). The leaf segment enclosed in each chamber was marked and photographed on a template, and its area was measured using ImageJ and then used to correct calculated gas exchange rates.

We used high ambient CO_2_ levels to ensure that photosynthesis was truly saturated by CO_2_, obviating the need to measure CO_2_ response curves to eliminate effects of varying stomatal conductance. This greatly increases throughput, but with two trade-offs. Firstly, it gives a value only for *A*_m_, and not more specifically for carboxylation capacity (*V*_cmax_) and electron transport capacity (*J*_max_). However, the ratio of *V*_cmax_ and *J*_max_ is highly conserved, both within and across taxa (Wullschleger 1993; Medlyn *et al.* 2002), so we reasoned that the roughly 10-fold increase in phenotyping time needed to complete CO_2_ response curves would not justify the likely small information gains in the present context. Secondly, photosynthesis is triose-phosphate-utilization (TPU)-limited at these high CO_2_ levels, necessitating validation to ensure that the assimilation rate under such conditions is a reliable proxy for the ‘true’ maximum value of *A*, which occurs at the transition between limitation of photosynthesis by RuBP regeneration and TPU. We validated our estimates of *A*_m_ by comparison with values inferred from *A* vs. *c*_i_ curves made on a subset of the leaves used in this study, and found high correspondence between the two values (*r*^2^ = 0.984; *n* = 18) (Salter *et al.* 2018a), indicating that *A*_m_ estimated by our procedure was a very reliable estimator of true *A*_m_.

Empty chamber tests revealed no significant diffusive leaks across our chamber gaskets (see Figure 4 in Salter *et al.* 2018a). We detected gasket leaks caused by imperfect sealing around leaf midribs by noting when chamber flow rate was greater with leaves in the chamber than without; in such cases we sealed the leak using clear silicone gap-filling compound. Leak sealing generally had no effect on calculated gas exchange rates, however, indicating that the leaks were predominantly advective and that the chamber air was thoroughly mixed.

Leaf temperature (*T*) was not controlled by OCTOflux. To minimize temperature fluctuations, the system was operated in an air-conditioned workshop. To correct *A*_m_ values to a common temperature of 25 °C, we determined the relationship between *A*_m_ and *T* (Salter *et al.* 2018a). Briefly, *A*_m_ was measured at three temperatures (21.1 ± 0.1, 26.1 ± 0.3 and 31.1 ± 0.05 °C) in each of 10 leaves using a calibrated IRGA (GFS-3000; Heinz Walz GmbH, Effeltrich, Germany). For each leaf, the function *A*_m_(*T*) = *a*·exp(*b*·*T*) was fitted to the data, *A*_m_ at 25 °C (*A*_m25_) was computed as *a*·exp(*b*·25) and each *A*_m_ value was expressed relative to its *A*_m25_ (*A*_rel_ = *A*_m_(*T*)/*A*_m25_). *A*_rel_ values were compiled across leaves for each temperature, the function *A*_rel_(*T*) = *a*′·exp(*b*′*·T*) was fitted to them (Figure 5 in Salter *et al.* 2018a) and this function was used to infer *A*_m25_ for each observed value of *A*_m_ in this study. Reported values of *A*_m_ herein are temperature-corrected to 25 °C.

### Measurement of daily irradiance

We used a quantum sensor (Li-190R, LI-COR) placed above the canopy to measure daily irradiance (*i*_d_, the integral of photosynthetically active photon flux, PPFD, over a day) above the canopy, and we used handmade ceptometers (‘PARbars’) placed between the flag and penultimate leaves, and below all leaves, to measure *i*_d_ above the penultimate leaf and below the canopy, respectively. The PARbars are described elsewhere (Salter *et al.* 2018b, 2019a). Briefly, they consist of 50 photodiodes (EAALSDY6444A0, Everlight Americas, Carrollton, TX, USA) attached to the underside of a white plastic diffuser bar (445 Opal White, Plastix Australia Pty Ltd, Arncliffe, NSW, Australia) at 2-cm intervals, with each contact soldered to a length of bare copper wire, all encased in epoxy (651 Universal Epoxy Potting Resin, Solid Solutions, East Bentleigh, VIC, Australia) for waterproofing and attached to a 1.25-m aluminum u-bar for rigidity. Each PARbar was calibrated against the quantum sensor immediately before the experiment began (Figure 3 in Salter *et al.* 2018b). PARbars were supported by 2.2-m aluminum square bars that spanned each plot and were supported at either end by gimbals attached to pipe clamps around PVC pipes held in position with sawhorses positioned in wheel tracks between plots (Figure 4 in Salter *et al.* 2018b). Bulls-eye levels placed atop each PARbar were used to level the support bars. The quantum sensor was placed atop a 1.6-m angle iron bar attached to a garden cart containing a datalogger (CR5000, Campbell Scientific, Logan, UT, USA), and levelled with a levelling mount. This arrangement enabled us to measure *i*_d_ in 34 plots simultaneously (two ranges of 17 rows) each day. The equipment was moved south-east to the next pair of ranges after sunset each day.

Photosynthetic photon flux density measured above the canopy will not generally be equal to that experienced by flag leaves, because flowering heads above the flag leaves intercept some light. To estimate light interception by heads, we placed PARbars above the flag leaves and below the heads in six plots, each of a different variety selected from the CSIRO lines grown in this study, grown in 2018 near Canberra. We calculated transmittance through the ‘head layer’ as the ratio of *i*_d_ measured by the PARbars below the heads to that measured by a quantum sensor above the heads, for 2 days in November 2018 (shortly after anthesis). Mean ± SE for head-layer transmittance was 0.841 ± 0.027. We subsequently calculated flag leaf daily irradiance (*i*_df_) as above-canopy PPFD multiplied by 0.841.

### Leaf area index and effective canopy extinction coefficient

We measured leaf area index (LAI) in each plot as follows. We harvested five tillers per plot within 3 days of the photosynthesis and ceptometry measurements made in that plot, measured the total area of all leaves on those tillers using a leaf area meter (Li-3100C, LI-COR) and divided the result by five to give average leaf area per tiller. We then measured the number of tillers per ground area in each plot by harvesting all tillers on a 1 m length of a single row, counting them and multiplying the result by the ratio of planting row length per plot (5 rows × 4 m length per row = 20 m) to area per plot (8 m^2^). Finally, we computed LAI as the product of total leaf area per tiller and number of tillers per ground area. We calculated canopy transmittance, *τ*_canopy_, as the ratio of *i*_d_(bottom) to *i*_d_(top) (*i*_d_ measured below and above the canopy, respectively), and computed the effective canopy extinction coefficient, *k*_canopy_, as ln[1/*τ*_canopy_]/LAI.

### Modeling effects of photosynthetic capacity redistribution on carbon gain

To quantify the increase in carbon gain if photosynthetic capacity were optimally redistributed between flag and penultimate leaves, we modelled daily carbon gain for flag and penultimate leaves of each genotype. Full details are provided in **Supporting Information—Appendix S1**, and summarized here. In one simulation, we computed daily carbon gain for each leaf based on measured photosynthetic capacities and daily irradiancies. In another simulation, we adjusted photosynthetic capacity in each leaf to maximize the sum of mean daily carbon gain for the two leaves combined, while holding total photosynthetic capacity constant. Each simulation comprised 55 time steps between sunrise and sunset. Photosynthetic photon flux density was computed for sunlit and shaded fractions of each leaf separately, as described by de Pury and Farquhar (1997), photosynthesis was calculated for each leaf fraction using the model of Farquhar *et al.* (1980) and leaf photosynthesis was computed as the weighted sum of the resulting values based on the sunlit fraction of leaf area (following de Pury and Farquhar 1997). Because we lacked genotype-specific data with which to parameterize a stomatal conductance model, we constrained the influence of stomata in our simulations by assuming that intercellular CO_2_ concentration (*c*_i_) was 280 ppm (70 % of ambient; Wong *et al.* 1979). Diurnal time courses for vapor-pressure deficit, wind speed and air temperature were modelled based on historical records for the study site, available from the Australian Bureau of Meteorology, and leaf temperature was estimated by energy balance. We performed each simulation twice, assuming cloudy or sunny conditions, and present results for both conditions. **Supporting Information—Figures S3****–****S5** provide sample time courses of assimilation rate, irradiance and meteorological conditions from these simulations.

### Leaf nitrogen and carbon isotope discrimination

Leaf segments used for gas exchange were sampled for nitrogen and carbon isotope analyses. Samples were oven-dried at 80 °C overnight. 1.3 mg of homogenously ground material was weighed into tin capsules (IVA Analysentechnik, Meerbusch, Germany) and inserted into a FlashHT modified to a dual reactor setup (reduction reactor at 680 °C and oxidation reactor at 1000 °C), coupled to a Delta V Advantage isotope ratio mass spectrometer (IRMS) by a Conflo IV interface (ThermoFisher Scientific, Bremen, Germany). δ ^13^C is expressed relative to VPDB (Vienna Pee Dee Belemnite). Internal standards with known nitrogen percentage (IVA Algal Standard: 1.25 %, proline: 12.17 % and L-glutamate: 9.52 %) and known isotopic composition were used, or were calibrated against primary isotope standards from the IAEA against VPDB for δ ^13^C: IAEA-CH-6 (−10.449‰) and Beet sucrose (−24.62‰). The precision of the analysis was below 0.12‰ for δ ^13^C and below 0.17 % for %N analysis.

### Statistical analysis

We tested for effects of leaf N and δ ^13^C on photosynthetic capacity using linear mixed models with genotype as a random effect, fitted using the lme4 package (Bates *et al.* 2015) in R and using function r2() in the sjstats package (Lüdecke 2020) to compute marginal *r*^2^ values for fixed effects. A linear mixed model was used to obtain best linear unbiased estimates (BLUE) of *A*_m2_, *A*_mf_, *A*_mf_/*A*_m2_ and *i*_f_/*i*_2._ All random effects were assumed to be normally and independently distributed and genotype was considered as both a fixed effect to estimate BLUE and as a random effect to estimate heritability as *σ*^2^_g_/(*σ*^2^_g_ + *ν*/2), where *σ*^2^_g_ is the variance component of genotype, and *ν* is the mean variance of a difference of two BLUE (Holland *et al.* 2002; Piepho and Möhring 2007). Polymorphic single-nucleotide polymorphisms (SNPs) were filtered using the PLINK software (http://pngu.mgh.harvard.edu/~purcell/plink/) to maintain SNPs with call rates greater than 40 % (Purcell *et al.* 2007; Anderson *et al.* 2010). Single-nucleotide polymorphisms without a map position were included and SNPs with a minor allele frequency (MAF) < 0.01 excluded from further analysis. Following filtering, 35 266 SNP markers were generated. The genome-wide association analysis (GWAS) was performed using the genome-wide complex analysis (GCTA) software (http://cnsgenomics.com/software/gcta/) following the procedure of Yang *et al.* (2011). The model fitted the overall mean (*μ*), fixed SNP effects and the genomic relationship matrix (GRM) to account for population structure. Thus, the model used to explain population structure was *y* = *μ* + SNP + random(GRM), where *y* represents population structure, *μ* the overall mean, SNP the fixed SNP effect and GRM the genomic relationship matrix. Following the linkage disequilibrium analysis those marker/trait associations with a −log10(*P*) value > 4 were retained as significant.

### Calculating the relative importance of variation in the components of *γ*

To identify the most important drivers of the observed variation in the coordination of photosynthetic capacity with light environment, as gauged by our phenotyping parameter *γ*, we used variance partitioning analysis as proposed by Rees *et al.* (2010). This method calculates the *relative importance* (*R*) of variation in each of several variables, *x*_1_, *x*_2_, ... *x*_*n*_, for driving variation in their sum, $\sum_{i=1}^{n}x_{i}$, as


$$\begin{array}{l} R(x_{i})=\frac{\overset{n}{\underset{j=1}{\sum }}\left|cov(x_{i},x_{j})\right|}{\overset{n}{\underset{i=1}{\sum }}\left(\overset{n}{\underset{j=1}{\sum }}\left|cov(x_{i},x_{j})\right|\right)} \end{array}$$ (1)

where |cov(*x*_*i*_, *x*_*j*_)| is the absolute value of the covariance of *x*_*i*_ and *x*_*j*_. In the present context, the variables of interest are the components of *γ*: *A*_mf_, *A*_m2_^−1^ and *i*_d2_/*i*_df_. The natural logarithms of these terms together add up to ln(*γ*). Thus, we applied Equation (1) to *x*_1_ = ln(*A*_mf_), *x*_2_ = ln(*A*_m2_^−1^) and *x*_3_ = ln(*i*_d2_/*i*_df_).

## Results

We measured *A*_m_ and daily irradiance (*i*_d_) in 160 genotypes of wheat. This included *A*_m_ in 1300 leaves—the flag and penultimate leaf on each of 650 individual tillers, for four tillers per genotype (two per plot, two plots per genotype)—and *i*_d_ above both flag and penultimate leaves and below all leaves in 320 plots (two per genotype). Because above-canopy *i*_d_ varied from day-to-day, we express all values relative to above-canopy *i*_d_, and thus report only the ratio of *i*_d_ between flag and penultimate leaves (*i*_df_/*i*_d2_). Original data for this ratio, as well as for flag- and penultimate leaf photosynthetic capacity, Zadoks score and yield, are provided for each genotype in Supporting Information—Table S2.

### Capacity per unit daily irradiance is systematically lower in flag leaves than in penultimate leaves

**Figure 1.**
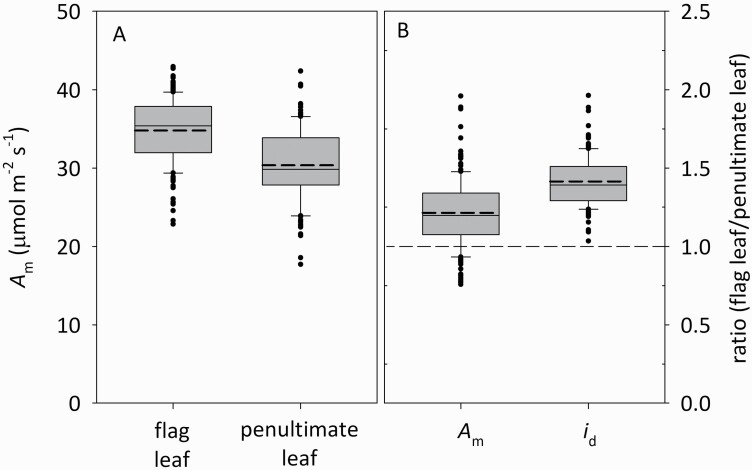
Distributions of (A) photosynthetic capacity (*A*_m_) in flag and penultimate leaves, and (B) the ratios of *A*_m_ and of daily irradiance (*i*_d_) between flag and penultimate leaves. Boxes denote the interquartile range (25th–75th percentile); whiskers denote the 10th and 90th percentiles; closed symbols denote outliers; and solid and dashed lines in the boxes denote medians and means, respectively. The dashed line across panel (B) indicates a value of 1.0. *n* = 160 genotypes.

**Figure 2.**
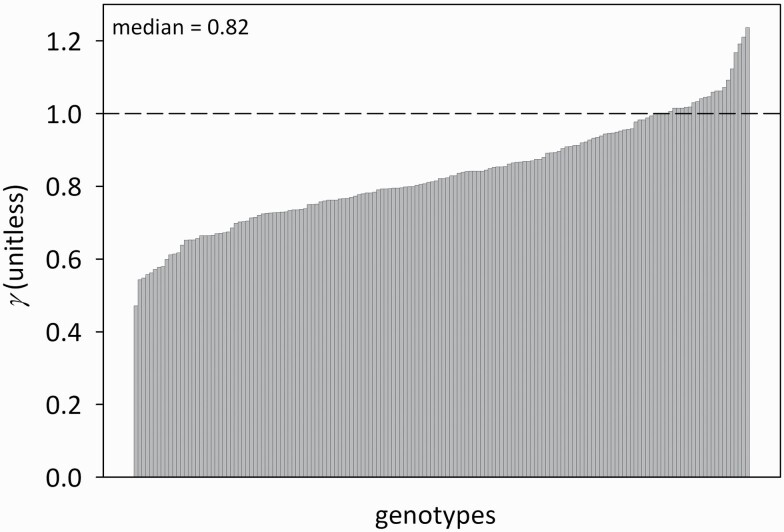
Distribution of *γ* (ratio of capacity per unit irradiance in flag leaf to that in penultimate leaf) across genotypes, ordered from smallest to largest value.

**Figure 3.**
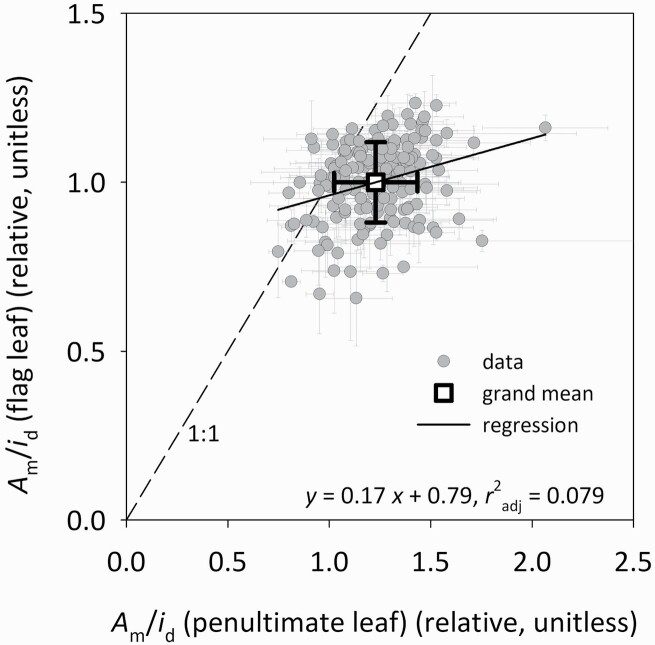
Photosynthetic capacity (*A*_m_) per unit daily irradiance (*i*_d_) in flag leaves (*y*-axis) vs. penultimate leaves (*x*-axis) across all genotypes (grey circles), both expressed relative to the grand mean in flag leaves (square white symbol). The dashed line is the 1:1 line; the solid line is a regression through the data; error bars are means ± SEs. *n* = 160 genotypes.

*A*_m_ was greater in flag leaves (*A*_mf_ = 34.79 ± 0.33 μmol m^−2^ s^−1^; mean ± SE) than in penultimate leaves (*A*_m2_ = 30.35 ± 0.37 μmol m^−2^ s^−1^) (Fig. 1; *F*(1,330) = 64.3, *P* < 0.001), and the two *A*_m_ values were positively but weakly correlated (*r*^2^ = 0.133, df = 648; **see****Supporting Information—Fig. S6**). The ratio of flag to penultimate leaf photosynthetic capacity (*A*_mf_/*A*_m2_) averaged 1.21 ± 0.02, but the ratio of daily irradiance in these two locations (*i*_df_/*i*_d2_) was greater, at 1.41 ± 0.01 (Fig. 1; *F*(1,330) = 168, *P* < 0.001). As a result, capacity per unit irradiance was smaller in flag leaves than in penultimate leaves, such that the ratio of capacity per unit irradiance between flag and penultimate leaves, or *γ* ([*A*_mf_/*i*_df_]/[*A*_m2_/*i*_d2_]) was less than unity, in 137 of 160 genotypes (Fig. 2), and *γ* differed significantly across genotypes (one-way ANOVA using *γ* calculated for each measured plant based on plot-level measurements of *i*_d2_/*i*_df_; *F*(165,486) = 1.23, *P* < 0.05). Capacity per unit irradiance was weakly correlated between flag and penultimate leaves in the same tiller (Fig. 3; capacity per unit irradiance [*A*_mf_/*i*_df_] = 0.17·[*A*_m2_/*i*_d2_] + 0.79, *r*^2^ = 0.079, *P* = 0.0002).

### Variation in canopy light penetration contributes minimally to variation in *γ*

Leaf area index varied among genotypes (mean ± SD: 2.39 ± 0.57 m^2^ m^−2^; **see****Supporting Information—Fig. S7**), as did canopy transmittance (*τ*_canopy_ = 0.286 ± 0.038; **see****Supporting Information—Fig. S7**; mean ± SD) and effective canopy extinction coefficient (*k*_canopy_ = 0.55 ± 0.13 m^2^ m^−2^; mean ± SD). *k*_canopy_ was uncorrelated with *γ***[see****Supporting Information—Fig. S8****]**, indicating that differences in the coordination of photosynthetic capacity with light were not strongly associated with differences in canopy structure that influence light penetration.

### Variation in *γ* is most strongly driven by variation in *A*_m_ in penultimate leaves

**Figure 4.**
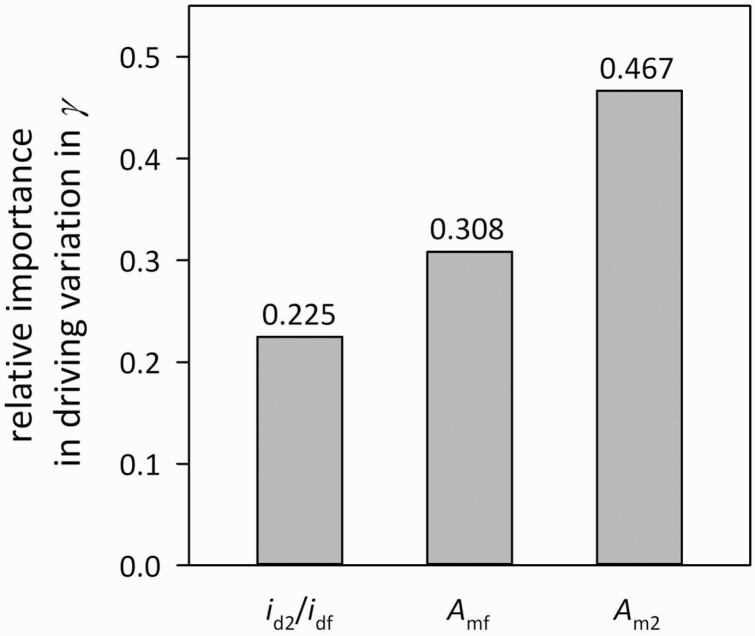
Relative importance of variation in the components of *γ* in driving its variation across genotypes, computed using the method of Rees *et al.* (2010). *i*_d2_/*i*_df_ is the ratio of daily irradiance between penultimate and flag leaves; *A*_mf_ is photosynthetic capacity in flag leaves; *A*_m2_ is photosynthetic capacity in penultimate leaves.

We used variance partitioning analysis (Rees *et al.* 2010) to quantify the relative importance of the components of *γ* in driving variation in *γ* across genotypes. We found that penultimate leaf photosynthetic capacity was by far the most important driver of variation in *γ* (relative importance [*R*] = 0.467)—more than twice as important as *i*_d2_/*i*_df_ (*R* = 0.225) and more than 1.5 times as important as flag leaf *A*_m_ (*R* = 0.308) (Fig. 4).

### Variation in *γ* is not driven by differences in phenology

Because the phenotyping campaign extended over 12 days, and also because genotypes may differ in flowering time and therefore in the timing of shifts in resource allocation among leaf layers, we also tested whether phenology (Zadoks stage, *Z*) contributed to *γ*, by regression analysis. *γ* was not significantly correlated with *Z* (*P* = 0.86, *r*^2^ = 0.0002; **see****Supporting Information—Fig. S9**). Excluding two genotypes outlying in *Z* on the basis of a significant two-sided Grubbs outlier test (*P* = 0.01), the correlation was even weaker (*P* = 0.99, *r*^2^ = 0.0000013).

### Preliminary marker-trait associations were discovered for penultimate leaf photosynthetic capacity

The heritability of photosynthetic capacity varied by trait, with the highest value of 0.425 observed for penultimate leaf photosynthetic capacity, *A*_m2_. The heritabilities of the remaining traits, including both *A*_mf_ and the composite traits *γ* and *A*_mf_/*A*_m2_, were less than 0.12, and these traits were not considered for further analysis. Genome-wide association analysis identified significant marker-trait associations for *A*_m2_ on six different chromosomes (Table 1; **see****Supporting Information—Fig. S10**). However, due to the relatively small number of genotypes represented in the analysis (118; the other genotypes could not be analysed due to IP restrictions), the associations of −log(*P*) > 4 will need to be confirmed in other materials and environments, and thus should be considered preliminary. Nevertheless, there are markers on chromosomes 1B, 6B and 7A, as well as several unmapped markers, that had a positive effect on *A*_m2_ that could potentially be targeted in plant breeding to improve canopy photosynthesis.

**Table 1.** Significant marker-trait associations for penultimate leaf photosynthetic capacity identified in a subset of materials evaluated in the field at Narrabri, NSW. Note that the last two characters in the Marker names (first column) should not necessarily be taken to imply a known chromosomal position; these unmapped markers were assigned to a hypothetical chromosome #8 in the Manhattan plot shown in **Supporting Information—Fig. S10**. NA indicates that the map position was not available.

Marker	Allele_frequency	Effect	−Log10(*P*)	Map position (bp)
77382_7A	0.0254	−7.223	4.856	1779249
66495_7B	0.9745	7.223	4.856	NA
25756_1B	0.9830	8.298	4.607	763007
48422_1B	0.9830	8.298	4.607	2338807
7806_1B	0.0169	−8.298	4.607	NA
47867_4A	0.9830	8.298	4.607	NA
65626_5A	0.0169	−8.298	4.607	NA
76596_6B	0.8135	4.565	4.200	2499243
77256_7A	0.9745	6.227	4.110	461240

### Optimal redistribution of photosynthetic N could increase canopy photosynthesis and its association with flag leaf photosynthetic capacity

**Figure 5.**
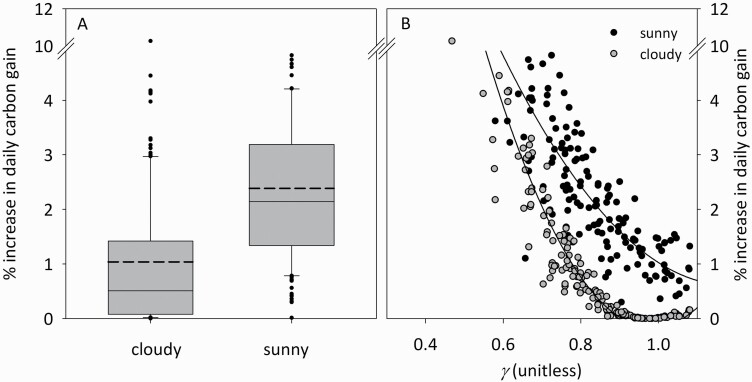
Predicted increase in the sum of daily mean net CO_2_ assimilation rates for flag and penultimate leaves combined, if photosynthetic capacity were optimally redistributed between the two leaves. Each point is one genotype. (A) Boxplots describing distribution of points shown in (B) (solid line in middle of box = median; dashed line = mean; box boundaries = 25th/75th percentiles; whisker bars = 10th/90th percentiles; closed symbols = outliers). Simulations for sunny and cloudy conditions used mean sunshine hours = 100 % or 0 %, respectively, of total daytime hours. Note the break in the y-axes between values of 4.99 and 10. *n* = 160 genotypes. (B) Effect of *γ* (ratio of photosynthetic capacity per unit of daily irradiance between flag and penultimate leaves) on predicted % gains; lines are logarithmic regression fits (sunny conditions [solid symbols]: %increase = 12.3·*γ*^2^ – 29.2·*γ* + 17.9, *r*^2^ = 0.76; cloudy conditions [grey symbols]: %increase = 27.6·*γ*^2^ – 54.3·*γ* + 26.5, *r*^2^ = 0.87).

**Figure 6.**
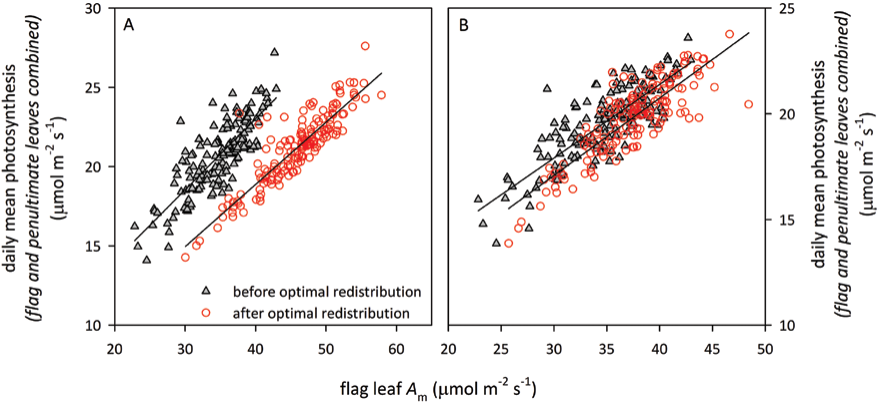
Simulated daily mean photosynthesis (sum of simulated daily average of net CO_2_ assimilation rates in flag and penultimate leaves) in relation to photosynthetic capacity of flag leaves, for (A) sunny, and (B) cloudy conditions, using the observed partitioning of photosynthetic capacity between flag and penultimate leaves (grey triangles), or with the same total capacity as observed, but re-distributed between the two leaves so as to maximize daily mean photosynthesis (open red circles). Solid lines are linear regressions (A: *r*^2^ = 0.67 [before] or 0.80 [after]; B: *r*^2^ = 0.67 [before], or 0.72 [after]). *n* = 160 genotypes.

Our simulations predicted that total daily carbon gain in the flag and penultimate leaves combined would increase by 0.7–5.0 % (sunny conditions; 5th–95th percentiles across genotypes, median = 2.1 %, mean = 2.4 % and maximum 8.0 %) or 0.0–4.0 % (cloudy conditions; median = 0.5 %, mean = 1.0 % and maximum 10.3 %) if photosynthetic N were redistributed between penultimate leaves and flag leaves so as to maximize the sum of daily mean assimilation rates at both positions (Fig. 5A). Differences in % gain across genotypes were strongly predicted by *γ* (*r*^2^ = 0.76 and 0.87 for sunny and cloudy conditions, respectively). % gain approached zero in genotypes with *γ* close to 1.0 (Fig. 5B). Before redistribution, a median of 57 % of total photosynthesis (flag + penultimate leaves) under sunny conditions occurred in flag leaves; after redistribution, this rose to 73.8 %. Moreover, before optimal redistribution, flag leaf photosynthetic capacity explained only 67 % of the variation in total photosynthesis for flag and penultimate leaves combined in sunny conditions, but after optimal redistribution, flag leaf *A*_m_ was a substantially better predictor of total photosynthesis, explaining 80 % of the variation (Fig. 6A). For cloudy conditions, however, the improvement was smaller (67 % before and 72 % after optimal redistribution) (Fig. 6B).

### Higher-throughput proxies were poor predictors of *A*_m_ and *γ*

Leaf N content was not significantly related to *A*_m_ in flag leaves, and was significantly but weakly related to *A*_m_ in penultimate leaves (*A*_m_/[μmol m^−2^ s^−1^] = 4.0·N/[g g^−1^] + 16.2; *r*^2^ = 0.12; *P* < 0.0001) and in both leaf classes combined (*A*_m_/[μmol m^−2^ s^−1^] = 2.6·N/[g g^−1^] + 22.8; *r*^2^ = 0.05; *P* < 0.005) **[see****Supporting Information—Fig. S11****]**. Similarly, the difference in δ ^13^C between flag and penultimate leaves was very weakly related to *γ* (*γ* = 0.584·[δ ^13^C_flag_ − δ ^13^C_penultimate_]/permille + 0.125, *r*^2^ = 0.033, *P* < 0.05) **[see****Supporting Information—Fig. S12****]**. Finally, yield was also unassociated with *γ* (*P* = 0.099, *r*^2^ = 0.017); excluding one low-yielding outlier on the basis of a significant Grubbs test (*P* = 0.01), the relationship weakened further (*P* = 0.18, *r*^2^ = 0.011) **[see****Supporting Information—Fig. S13****]**.

## Discussion

We phenotyped photosynthetic capacity (*A*_m_) in 160 genotypes of wheat—a wider range of genetic diversity than previously examined for this trait in wheat, to our knowledge (cf. 108 accessions (Carver and Nevo 1990) and 64 genotypes (Driever *et al.* 2014)), or in any species grown under field conditions (cf. 215 rice genotypes grown in pots (Qu *et al.* 2017)). We also phenotyped daily irradiance with spatially integrating ceptometers at two canopy positions across all genotypes, and developed a useful diagnostic for suboptimal capacity distribution, *γ* (the ratio of photosynthetic capacity per unit daily irradiance in flag and penultimate leaves). Our results extend and clarify earlier reports of poor coordination between photosynthetic capacity and the local light environment (e.g. Hirose and Werger 1987, 1994; Hollinger 1996; de Pury and Farquhar 1997; Makino *et al.* 1997; Bond and Kavanagh 1999; Friend 2001; Frak *et al.* 2002; Lloyd *et al.* 2010; Townsend *et al.* 2018) by showing, for the first time, that the degree of coordination varies widely across genotypes. Our modeling predicts that harnessing this variation through directed breeding for increased *γ* could enhance total photosynthesis in flag and penultimate leaves combined by up to 5 % in sunny environments.

### Potential for improved canopy photosynthesis to enhance yield

Previous studies have found either a weak correspondence between yield and flag leaf photosynthetic capacity (e.g. Driever *et al.* 2014), or that such a correspondence only exists under abiotic stress (Lopes and Reynolds 2012). This has been interpreted as evidence that yield is not limited by supply of reduced carbon from the canopy, and that research should therefore focus on improving sink strength rather than photosynthesis (Smith *et al.* 2018). Our data and data-driven modelling offer a subtly different interpretation: flag leaf *A*_m_ is a poor predictor of yield in part because it is a poor predictor of canopy carbon gain, which in turn is a consequence of suboptimal distribution of photosynthetic capacity in the canopy. For observed capacity profiles, flag leaf *A*_m_ predicted only 67 % of variance in total photosynthesis of flag and penultimate leaves combined, leaving 33 % of variance unexplained (Fig. 6). However, our model predicted that the unexplained variance would drop by nearly half, to 20 %, if photosynthetic capacity were optimally redistributed from penultimate to flag leaves for plants in sunny environments (Fig. 6A). This suggests that phenotyping for photosynthetic traits is substantially more informative when combined with phenotyping for spatial coordination of photosynthetic potential with irradiance. It also parallels recent evidence that canopy photosynthesis in wheat is substantially reduced by suboptimal N partitioning between lower and upper canopy layers (Townsend *et al.* 2018), and that yield can also be enhanced by improving the *temporal* coordination of photosynthetic potential with irradiance (Kromdijk *et al.* 2016; Taylor and Long 2017; Salter *et al.* 2019b).

Our phenotyping parameter, *γ*, strongly predicted the potential for redistribution of photosynthetic capacity to increase total photosynthesis. This parameter can thus inform breeding efforts in several ways. Increasing carbon gain by modifying genotypes with already high yield and good agronomic characteristics, but with low *γ*, could increase yields within the constraints of existing agronomic limitations on N application. This can be achieved either by using the markers tentatively identified here for traits controlling *γ* (after further validation of those markers), or by using genotypes with high *γ* as genetic source material for crossing. Further pre-breeding advancements could also arise from intensive study of genotypes with contrasting *γ* to determine the underlying physiological and genetic mechanisms. A challenge facing any attempt to harness variation in *γ* will be to phenotype this parameter in large breeding populations. Our direct approach would be impractical for large populations with many hundreds of lines. Higher-throughput proxies could be helpful; for example, leaf N content could substitute for *A*_m_, and differences in capacity per unit irradiance between canopy layers might be reflected in differences in carbon isotope discrimination (δ ^13^C). However, we found poor correspondence between *A*_m_ and leaf N **[see****Supporting Information—Fig. S9****]**, and between *γ* and the difference in δ ^13^C between flag and penultimate leaves **[see****Supporting Information—Fig. S10****]**. The development of higher-throughput methods to estimate *γ* is an important goal for future research.

Our findings do not discount the importance of sink strength. Indeed, the activity of sink tissues and their effect on photosynthesis remain poorly studied, in part due to a lack of phenotyping methods that can measure sink development at appropriate scales (for reviews, see Paul and Foyer 2001; White *et al.* 2016; Smith *et al.* 2018). As a result, the potential benefit of enhancing sink strength through breeding or genetic manipulation is largely unknown. Yet, just as enhancements in canopy photosynthesis make sink strength the main limiting factor, any enhancements in sink strength would shift the dominant limitation back to source strength. Thus, concurrent research to improve both source and sink strength, as well as the coordination between the two, is clearly warranted. Our results emphasize the importance of considering *canopy-scale* source strength rather than single-leaf photosynthesis, as noted previously (Richards 2000; Long *et al.* 2006; Evans 2013; Furbank *et al.* 2015; Long *et al.* 2015; Wu *et al.* 2016).

### Variation in *γ* is driven largely by variation in penultimate leaf photosynthetic capacity, not canopy light penetration

The importance of considering canopy-scale photosynthesis in addition to single-leaf photosynthesis has long been acknowledged (Wells *et al.* 1982; Zelitch 1982; Townsend *et al.* 2018). However, in the context of crop improvement, most attention to this distinction has focused on canopy architecture: i.e. selecting for architectural traits that deliver more light to the lower canopy (e.g. Carvalho and Qualset 1978; Araus *et al.* 1993; Song *et al.* 2013). Yet we found little evidence that canopies with greater light penetration to lower layers (as gauged by a smaller effective canopy light extinction coefficient, *k*_canopy_) had more nearly optimal distributions of photosynthetic capacity (larger *γ*) **[see****Supporting Information—Fig. S8****]**. We found instead that the strongest driver of variation in *γ* was the photosynthetic capacity of penultimate leaves, with *A*_m_ in flag leaves a distant second and light penetration to penultimate leaves the least important (Fig. 4). The particular dependence of *γ* on penultimate leaf *A*_m_ may reflect genotypic variation in the tendency or capacity to re-translocate N from penultimate to flag leaves when the former become shaded by the latter as flag leaves develop. This hypothesis could be tested by directly tracking penultimate and flag leaf *A*_m_, Rubisco and N content, and translocation during canopy development, in both low- and high-*γ* genotypes. Reducing recalcitrance of photosynthetic N in penultimate leaves through selection would also increase availability of N for grain filling. In this context, it is important to consider the possibility that genotype differences in coordination of *A*_m_ between penultimate and flag leaves could, in principle, arise due to genotype differences in developmental timing; however, we recorded developmental stages (Zadok score, *Z*) for every measured tiller and found no relationship between *γ* and *Z*.

An alternative hypothesis to explain the apparent importance of penultimate leaf *A*_m_ is that flag leaf *A*_m_ is somehow constrained, either by (i) a practical upper limit on the magnitude of leaf N content and/or leaf mass per unit area (Meir *et al.* 2002; Lloyd *et al.* 2010; Dewar *et al.* 2012), or (ii) a tendency for N to be diverted to developing heads during flag leaf development. Either mechanism could make theoretically optimal *A*_m_ values impossible to achieve in flag leaves. Some evidence does indicate that leaf N content is genetically constrained (Fyllas *et al.* 2009), though the magnitudes of flag leaf *A*_m_ reported here are not especially large (mean 35 μmol m^−2^ s^−1^, 90th percentile 38 μmol m^−2^ s^−1^): wheat flag leaf *A*_m_ is commonly well over 40 μmol m^−2^ s^−1^ (Watanabe *et al.* 1994; Driever *et al.* 2014; Taylor and Long 2017). Regarding hypothesis (ii), heads do become a strong N sink early in development (Rao and Dao 1996), largely coincident with flag leaf development. This hypothesis could be tested by observing whether *γ* increases if competition for N between heads and flag leaves is reduced by increasing soil N supply during head and flag leaf development.

### Limitations to this study

The generality of our conclusions may be limited by three factors. First, we did not test whether flag and penultimate leaves differed systematically in orientation across genotypes, which could cause their effective incident irradiances to differ from those we measured using levelled ceptometers. If penultimate leaves were systematically more nearly level than flag leaves, then our estimates of *γ* would be too low, indicating a smaller degree of suboptimality than suggested by our measurements. Second, we did not measure light absorption by heads in each genotype, but instead estimated this using an average value from six genotypes. Systematic correlation between head light capture and light penetration between flag and penultimate leaves could invalidate our results, and should be assessed in future work. Third, logistical constraints led us to use a row planter with 40 cm row spacing, which is wider than typical in Australia and likely increased light penetration to penultimate leaves. A more typical row spacing would likely lead to lower irradiance in penultimate leaves, and if anything, lower values of *γ* than we observed, suggesting that the genetic variation that we uncovered in this study in the coordination of *A*_m_ with light environment is likely to have an even greater influence on canopy-level photosynthetic performance than indicated by our simulations.

## Conclusions

The present study has shown that genetic variation exists in the coordination of photosynthetic capacity with local light environment within wheat canopies, and that total photosynthesis for flag and penultimate leaves combined could be increased by harnessing this variation through directed breeding. We characterised a novel metric (*γ*) to quantify deviations from optimal N partitioning, and our preliminary GWAS identified several molecular markers potentially associated with traits governing variation in *γ*. Our modeling predicts that potential increases in canopy-scale carbon capture are significant and would contribute to increased yield potential. Our results also support recent evidence that efforts to improve crop photosynthesis must look beyond the flag leaf, and consider heterogeneity within the canopy.

## Supporting Information

The following additional information is available in the online version of this article—

**Figure S1.** Field plot layout.

**Figure S2.** Zadoks stages.

**Figure S3.** Sample photosynthesis simulations.

**Figure S4.** Sample irradiance simulations.

**Figure S5.** Sample time courses of meteorological conditions.

**Figure S6.** Flag vs. penultimate leaf photosynthetic capacity.

**Figure S7.** Leaf area index (LAI), canopy transmittance and effective canopy extinction coefficient.

**Figure S8.***γ* vs. *k*_canopy_.

**Figure S9.***γ* vs. Zadoks score.

**Figure S10.** Manhattan plot for genome-wide association analysis (GWAS) results.

**Figure S11.** Photosynthetic capacity vs. leaf N content.

**Figure S12.** Difference in δ ^13^C vs. *γ*.

**Figure S13.** Grain yield vs. *γ*.

**Table S1.** List of genotypes.

**Table S2.***γ* and its components, Zadoks score and yield for 160 genotypes.

**Appendix S1.** Explanation of simulation methods.

plaa039_suppl_Supplementary_Figure_S1_S12

plaa039_suppl_Supplementary_Table_S1

plaa039_suppl_Supplementary_Table_S2
