# Supplementary material for: Wide variation in the suboptimal distribution of photosynthetic capacity in relation to light across genotypes of wheat
Source: AoB Plants. 2020 Aug 11;12(5):plaa039. doi: 10.1093/aobpla/plaa039 (PMC7494244; doi:10.1093/aobpla/plaa039)
Supplement: plaa039_suppl_Supplementary_Figure_S1_S12 [file plaa039_suppl_supplementary_figure_s1_s12.pdf]

Supplementary File S1

" Wide variation in the suboptimal distribution of photosynthetic capacity in relation to light across genotypes of wheat," by William T Salter, Andrew Merchant, Richard M Trethowan, Richard A Richards and Thomas N Buckley

Contents:

Figure S1. Field plot layout.
Figure S2. Zadoks stages.
Appendix S1. Explanation of simulation methods.
Figure S3. Sample photosynthesis simulations.
Figure S4. Sample irradiance simulations.
Figure S5. Sample timecourses of meteorological conditions.
Figure S6. Flag vs penultimate leaf photosynthetic capacity.
Figure S7. LAI, canopy transmittance and effective canopy extinction coefficient.
Figure S8. γ vs k_{canopy}.
Figure S9. γ vs Zadoks score.
Figure S10. Manhattan plot for GWAS results.
Figure S11. Photosynthetic capacity vs leaf N content.
Figure S12. Difference in $\delta^{13}C$ vs. γ.
Figure S13. Grain yield vs γ.

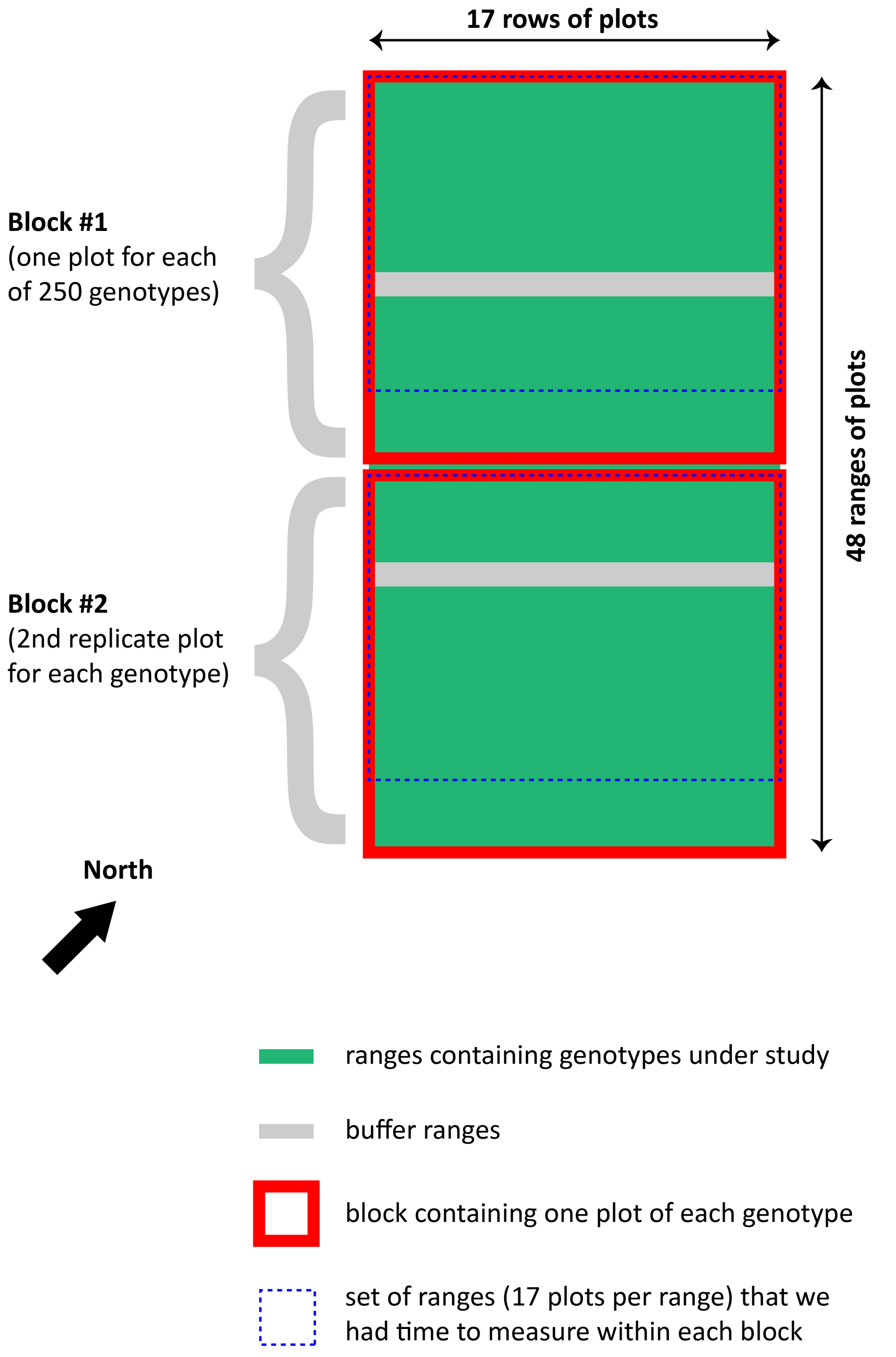

Figure S1. Diagram of layout of field plots for the experiment described in this study.

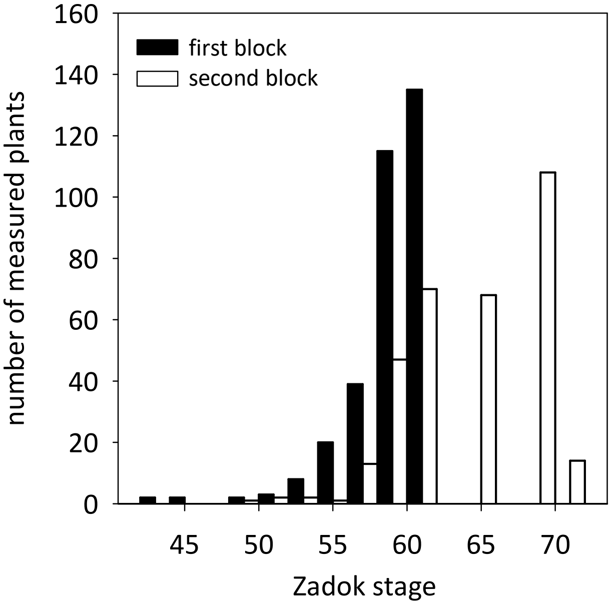

Figure S2. Distribution of phenological stages (Zadok stages) of plants measured in this study. First block = 03 – 10 Sep 2017, second block = 11 – 18 Sep 2017.

Appendix S1. Simulations to estimate optimal redistribution of photosynthetic N between penultimate and flag leaves

A. Overview

We simulated daily photosynthesis in both penultimate and flag leaves of each genotype, using the observed values of photosynthetic capacity (A_m) in both leaf layers, and again, using values of A_m adjusted so as to maximize the total photosynthesis in both layers combined, but while holding total N constant.

B. Photosynthesis model

We used the photosynthesis model of Farquhar et al (1980) to simulate photosynthesis separately for each leaf layer (flag and penultimate) in each genotype, and for sunlit and shaded fractions of each layer, and summed the sunlit and shaded values using expressions given by de Pury and Farquhar (1997) to give total daily photosynthesis for each layer. Net CO_2 assimilation rate (A, μmol m^{-2} s^{-1}) is calculated as the lesser of two values, one (A_v) limited by RuBP carboxylation and another (A_j) limited by RuBP regeneration:

(S1) $$A = \min\{A_v, A_j\}$$

(S2) $$A_v = V_m\left(\frac{c_i - \Gamma^*}{c_i + K'}\right) - R_d$$

(S3) $$A_j = \frac{1}{4}J\left(\frac{c_i - \Gamma^*}{c_i + 2\Gamma^*}\right) - R_d$$

where V_m (μmol m^{-2} s^{-1}) is carboxylation capacity (maximum RuBP carboxylation velocity), c_i (μmol mol^{-1}) is the intercellular CO_2 concentration, Γ^* (μmol mol^{-1}) is photorespiratory CO_2 compensation point, K' (μmol mol^{-1}) is the effective Michaelis constant for RuBP carboxylation, R_d (μmol m^{-2} s^{-1}) is the rate of non-photorespiratory CO_2 release that continues in the light, and J (μmol m^{-2} s^{-1}) is the potential electron transport rate. We assumed that c_i = 280 ppm (~70% of ambient; Wong *et al.*, 1979), and calculated Γ^*, K' and R_d from expressions given by de Pury and Farquhar (1997), based on leaf temperature (estimated as described in section F below). We calculated J as the lesser root of the expression

(S4) $$0 = \theta_j J^2 - J(J_m + \phi i) + J_m \phi i$$

where θ_j is a dimensionless curvature factor (assumed to be 0.7), i (μmol m^{-2} s^{-1}) is incident PPFD, and ϕ is the initial slope of J vs i (assumed to be 0.3 μmol μmol^{-1}).

C. Calculating photosynthetic parameters that depend on nitrogen (V_m, J_m and R_d)

We assumed that total photosynthetic N for each layer was the sum of components that determine and are each proportional to carboxylation and electron transport capacities (V_m and J_m, respectively), that J_m = 2.1 V_m (Wullschleger, 1993; Medlyn *et al.*, 2002), that $A_m = 0.25 \cdot J - R_d$ (the limit of Eqn S3 at high c_i), and that $R_d = 0.0089 \cdot V_m$ (de Pury and Farquhar, 1997). Solving Eqn S4 for J_m and applying θ_j = 0.7, ϕ = 0.3 and i = 1700 μmol m^{-2} s^{-1} (the value during measurements of A_m) gives

(S5) $$J_m = J\left(\frac{0.3 \cdot 1700 - 0.7 \cdot J}{0.3 \cdot 1700 - J}\right)$$

Since $R_d = 0.0089 \cdot V_m = 0.0089 \cdot J_m/2.1 = 0.00424 \cdot J_m$, and $A_m = 0.25 \cdot J - R_d$, it follows that

(S6) $$A_m = \frac{1}{4}J - 0.00424 \cdot J\left(\frac{0.3 \cdot 1700 - 0.7 \cdot J}{0.3 \cdot 1700 - J}\right)$$

Eqn S6 leads to a quadratic expression for J, which can be solved for J and the result applied to S5 to give J_m.

The assumptions outlined above imply that if total N between both leaf layers is conserved, then the sum of J_m in both layers is also conserved. Thus, for each genotype, we calculated initial J_m in each layer from A_m using Eqns S5 and S6 as described above, and then calculated their sum ($J_{m,total}$). To maximize total photosynthesis for both leaves combined while ensuring total N for both layers was conserved, we numerically adjusted "final" (post-redistribution) flag leaf J_m (J_{mf}') and calculated final J_m for the penultimate leaf (J_{m2}') as $J_{m2}' = J_{m,total} - J_{mf}'$. Finally, we calculated V_m and R_d in each layer for both pre- and post-redistribution conditions as $J_m/2.1$ and $0.0089 \cdot V_m$, respectively. J_m, V_m and R_d were corrected for temperature as described by de Pury and Farquhar (de Pury and Farquhar, 1997).

Simulated diurnal timecourses of net CO_2 assimilation rate are shown in Figure S2.

D. Simulating incident PPFD (i)

In each leaf layer, we computed the daily timecourse of PPFD in both sunlit and shaded fractions separately, using expressions given by de Pury and Farquhar (1997), with a timestep of 0.2 hours, from sunrise to sunset (~6:36 am – 5:24 pm; 55 total timesteps). Those expressions take as an input the cumulative leaf area index (L, $m^2\ m^{-2}$) above the layer in question. To calculate L for the flag leaf layer, we numerically adjusted L until the simulated total daily irradiance was 0.841 times the above-canopy value (0.841 was the mean ratio of daily irradiance above the flag leaf to that above the canopy, measured in the field). To calculate L for the penultimate leaf layer, we adjusted L until the ratio of simulated daily irradiance between the penultimate and flag leaf layers was equal to the ratio measured in the field by ceptometry for the genotype in question (that ratio was denoted as i_{d2}/i_{df} in the main text).

The canopy light model also required a value for atmospheric transmissivity (τ_a, dimensionless), which influences the distribution of incoming light between beam (direct) and scattered (diffuse) components. We used expressions of Roderick (1999), which predict the diffuse fraction of total irradiance (f_d, dimensionless) as a function of the ratio of sunshine hours to total daytime hours, or equivalently, the sunshine probability (p_{sun}, dimensionless), given the latitude. Those expressions predict that for our study site (Narrabri, NSW, -30.283° latitude), $f_d = 0.1513$ for $p_{sun} = 1.0$ (sunny conditions), and $f_d = 0.960$ for $p_{sun} = 0.0$ (cloudy conditions). We adjusted τ_a iteratively to make simulated daily diffuse irradiance as a fraction of total daily irradiance (above the canopy) match these values of f_d, giving $\tau_a = 0.076$ (cloudy) and 0.830 (sunny).

Finally, the canopy light model also required input values for day of the year (254 = 11 September 2017, the middle of our measurement campaign), a longitudinal correction for solar noon (-0.023 hours at Narrabri), and latitude (-30.283°). Sample timecourses for incident irradiance are shown in Figure S3.

E. Simulating other environmental parameters

We simulated diurnal trends for other climatic conditions using historical climate records for a weather station near our study site, available from the Australian Bureau of Meteorology (Narrabri West Post Office, site #053030; data available at bom.gov.au). We based our simulations on 9 am

and 3 pm mean values for air temperature in early September (T_a: 14.56 and 21.7°C, respectively), air relative humidity (h: 67 and 39.75%, respectively) and wind speed (v_w: 15.5 and 19.4 km h^{-1}, respectively), and also the mean daily minimum and maximum air temperatures (T_{amin} = 6.85°C and T_{amax} = 22.5 °C, respectively). We assumed that T_a varied sinusoidally during the day, according to the function $T_a = T_{amin} + (T_{amax} - T_{amin})\cdot 0.5\cdot(1 - \cos(\pi\cdot(t - t_o)/t_s))$, where π = 3.1415..., t is time of day (hours) and t_o and t_s are empirical parameters (both hours); and we adjusted t_o and t_s iteratively to match the reported mean 9 am and 3 pm values (giving t_o = 4.417 and t_s = 9.241 hours). We assumed that the absolute vapor pressure (p_{wa}, Pa) varied linearly with time ($p_{wa} = p_{wao} + m_{wa}\cdot t$, where p_{wao} and m_{wa} are empirical parameters), and adjusted p_{wao} and m_{wa} to match the reported mean values of relative humidity ($h(t) = 100\cdot p_{wa}(t)/p_{sat}(T_a(t))$, where p_{sat} is the saturation vapor pressure, given by $p_{sat}(T) = 611.2\cdot\exp(17.62\cdot T/(243.12 + T))$, with T in °C and p_{sat} in Pa) at 9 am and 3 pm (giving p_{wao} = 1229 Pa and m_{wa} = -13.33 Pa h^{-1}). We assumed that the wind speed varied linearly with time ($v_w = v_{wo} + m_{vw}\cdot t$, where v_{wo} and m_{vw} are empirical parameers), and adjusted v_{wo} and m_{vw} to match the reported mean values at 9 am and 3 pm (giving v_{wo} = 9.65 km h^{-1} and m_{vw} = 0.65 km h^{-2}). From p_{wa} and T_a we calculated the vapor pressure deficit of the air, $D_{air}(t)$, as $p_{sat}(T_a(t)) - p_{wa}(t)$.

Simulated timecourses of T_a, VPD and v_w are shown in Figure S4.

<u>*F. Simulating leaf temperature*</u>

We simulated leaf temperature in each leaf layer by energy balance:

$$0 = Q + f_{ir}\varepsilon_a\sigma T_{aK}^4 - f_{ir}\varepsilon_L\sigma T_{LK}^4 - g_{bh}c_{pa}(T_L - T_a) - \lambda g_{tw}D_{leaf}/p_{atm} \quad \text{(S7)}$$

where Q (J m^{-2} s^{-1}) is absorbed shortwave radiation (0.3173*i; this accounts for absorption of both visible and near-infrared radiation; Buckley *et al.*, 2014), f_{ir} (unitless) is the fraction of possible infrared exchange that occurs between the leaf in question and the atmosphere, ε_a (unitless) is the atmospheric IR emissivity, ε_L is the leaf IR emissivity (0.98), T_{aK} is air temperature in kelvins, T_{LK} is leaf temperature in kelvins, g_{bh} (mol m^{-2} s^{-1}) is the (2-sided) leaf boundary layer conductance, c_{pa} is the molar heat capacity of the air (29.3 J mol^{-1} K^{-1}), T_L is leaf temperature in °C, λ is the latent heat of vaporization (4.4·10^4 J mol^{-1}), g_{tw} (mol m^{-2} s^{-1}) is total leaf conductance to water vapor, D_{leaf} (Pa) is the vapor pressure difference between the leaf intercellular airspaces and the air, and p_{atm} (Pa) is atmospheric pressure (9.89·10^4 Pa in Narrabri [212 m elevation]). Eqn S7 ignores metabolic energy storage, assumes zero net IR exchange among leaves in the canopy, and assumes stomatal conductances are similar at both leaf surfaces. We assumed that the canopy attenuates IR with an extinction coefficient equal to that for diffuse shortwave radiation (kd' = 0.719; de Pury and Farquhar, 1997), so that $f_{ir} = \exp(-k_d'\cdot L)$. ε_a is a function of air temperature and vapor pressure ($\varepsilon_a = 0.642\cdot(p_{wa}/T_{aK})^{1/7}$) (Leuning *et al.*, 1995). We modeled g_{bh} as a function of leaf width (assumed to be 2 cm) and wind speed ($g_{bh} = 2\cdot 0.123\cdot(v_w(L)/0.02\text{ m})^{1/2}$ for v_w in m s^{-1}; ref), and assumed that wind speed attenuated down through the canopy with an extinction coefficient of 0.5, such that $v_w(L) = v_w(0)\cdot\exp(-0.5\cdot L)$, where $v_w(0)$ is the value above the canopy, calculated as described in section E. We calculated g_{tw} as $(r_{sw} + r_{bw})^{-1}$ (where r_{sw} and r_{bw} are stomatal and boundary layer resistances to water vapor, respectively), and computed r_{sw} as $(1/1.6)\cdot(r_{tc} - r_{bc})$ (where r_{tc} and r_{bc} are total and boundary layer resistances for CO_2, respectively) and r_{bw} as $r_{bc}/1.37$. $r_{tc} = A/(400 - c_i)$ and $r_{bc} = 1.27/g_{bh}$. Together this gives $g_{tw} = 1.6/(A/(400 - c_i) + 0.213/g_{bh})$.

D_{leaf} depends on leaf temperature, but can be estimated from D_{air} by linear extrapolation: $D_{leaf} \approx D_{air} + s\cdot(T_L - T_a)$, where $s = \partial p_{sat}/\partial T = 17.62\cdot 243.12/(243.12+T)^2$. Similarly T_{LK}^4 can be approximated by expansion of $(T_{aK} + [T_L - T_a])^4 \approx T_{aK}^4 + 4\cdot T_{aK}^3\cdot(T_L - T_a)$. Applying these approximations to Eqn S7 and rearranging leads to the following expression for T_L:

(S8) $$T_L = T_a + \frac{0.3173 \cdot i + f_{ir}(\varepsilon_a - \varepsilon_L)\sigma T_{aK}^4 - \lambda g_{tw} D_{air}/p_{atm}}{4 f_{ir} \varepsilon_L \sigma T_{aK}^3 + g_{bh} c_{pa} + \lambda g_{tw} s/p_{atm}}$$

Since the estimate of T_L given by Eqn S8 depends on T_L itself via A (from which g_{tw} is estimated), we used an iterative procedure: first using a leaf temperature of $T_{L,1}$ = 25°C to calculate A_1, $g_{tw,1}$ from A_1, and $T_{L,2}$ from $g_{tw,2}$; then calculating A_2 from $T_{L,2}$, $g_{tw,2}$ from A_2, and $T_{L,3}$ from $g_{tw,2}$; and finally recalculating a final value of A from $T_{L,3}$.

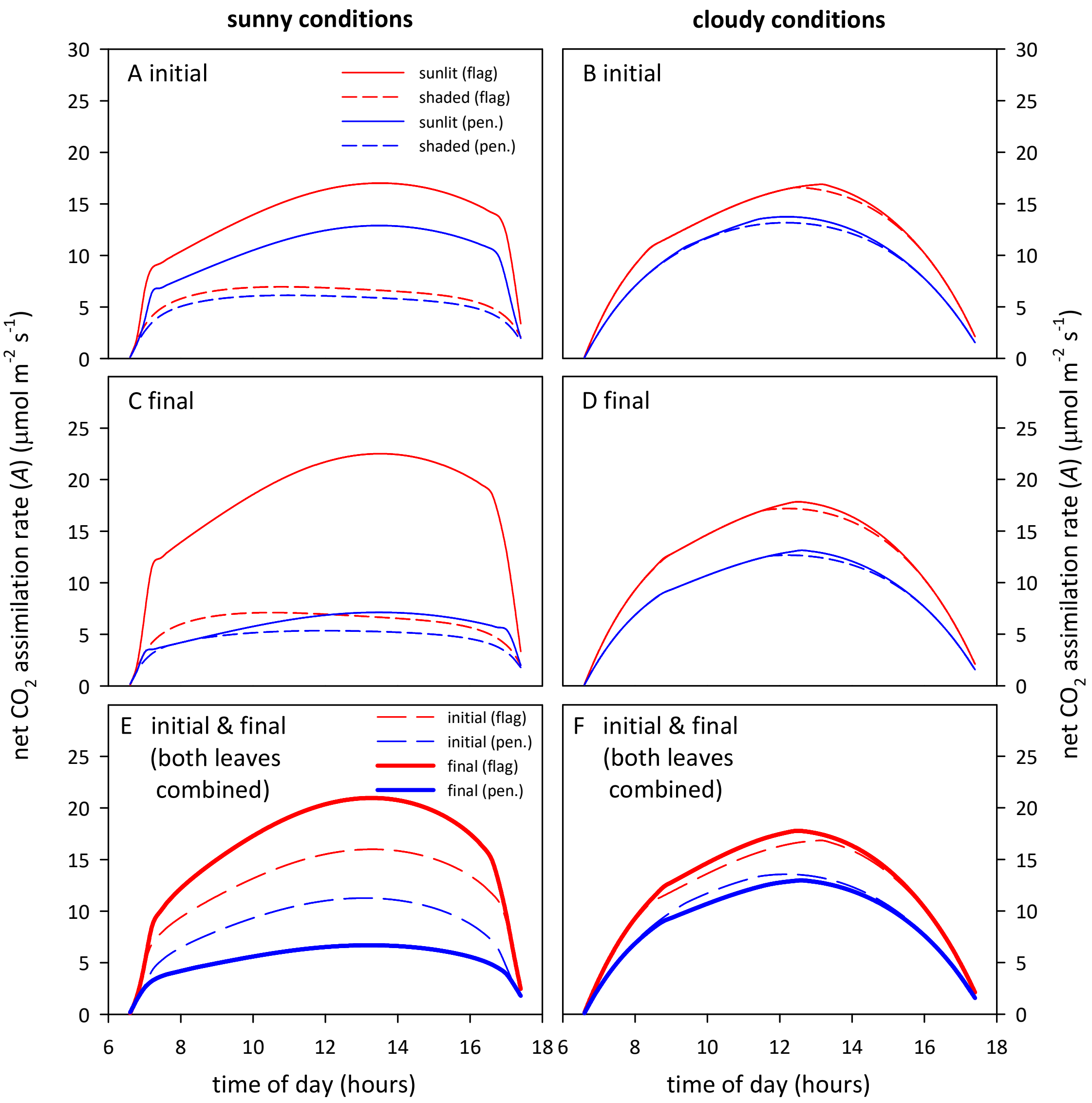

Figure S3. Sample simulations of net CO_2 assimilation rate in (a-d) the shaded and sunlit fractions of flag and penultimate leaves, under both (A,C) sunny and (B,D) cloudy conditions, before (A,B) and after (C,D) optimal redistribution of photosynthetic capacity. (E,F) Whole-layer photosynthesis (sunlit and shaded fractions of the layer combined) before and after redistribution. Simulations for (A,C,E) and (B,D,F) used data from the genotypes with % gain from redistribution closest to the medians for sunny and cloudy conditions, respectively. These genotypes were also used for Figure S4.

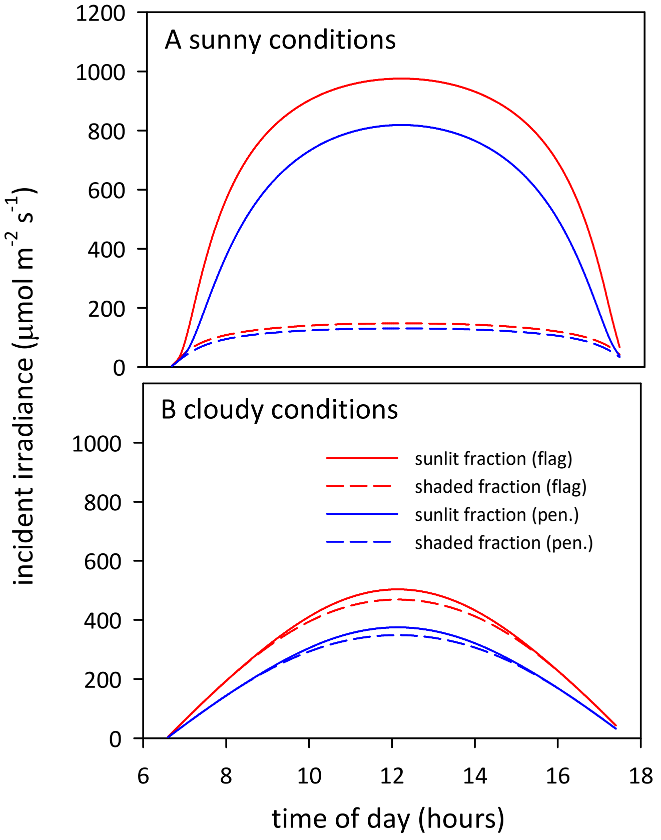

Figure S4. Sample simulations of incident PPFD in the shaded (solid lines) and sunlit fractions (dashed lines) of flag (red lines) and penultimate leaves (blue lines, "pen."), under both (A) sunny and (B) cloudy conditions. Simulations shown here are for the same leaves as shown in Figure S3.

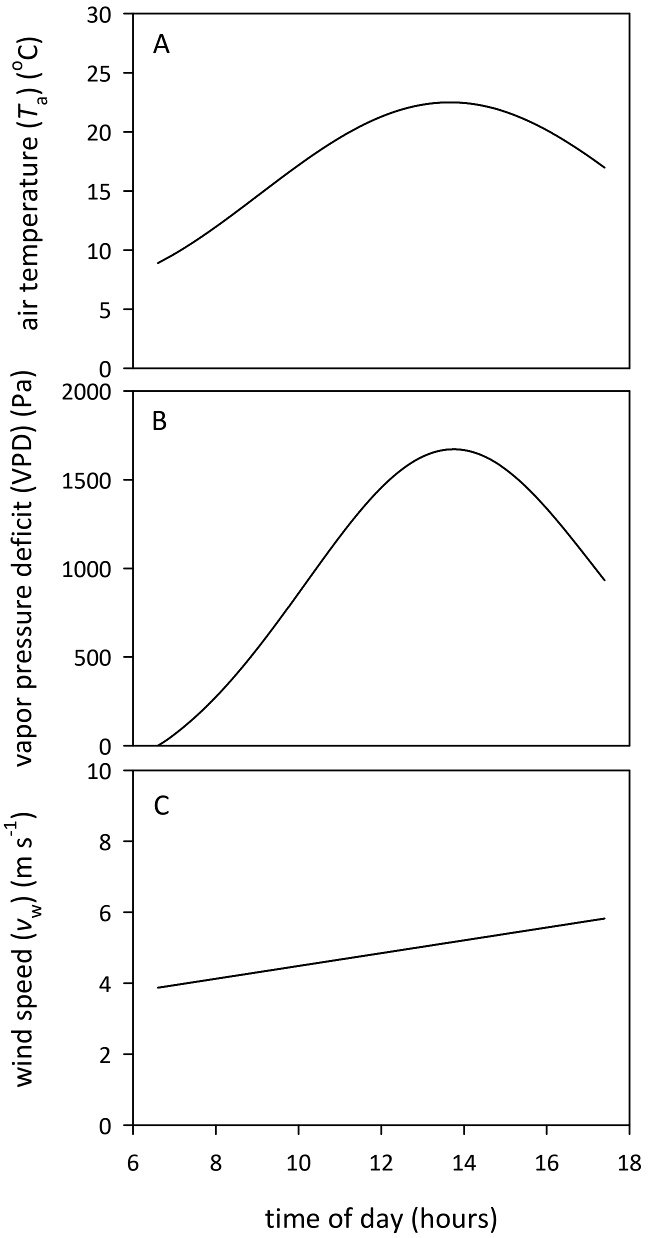

Figure S5. Simulated diurnal timecourses of environmental conditions.

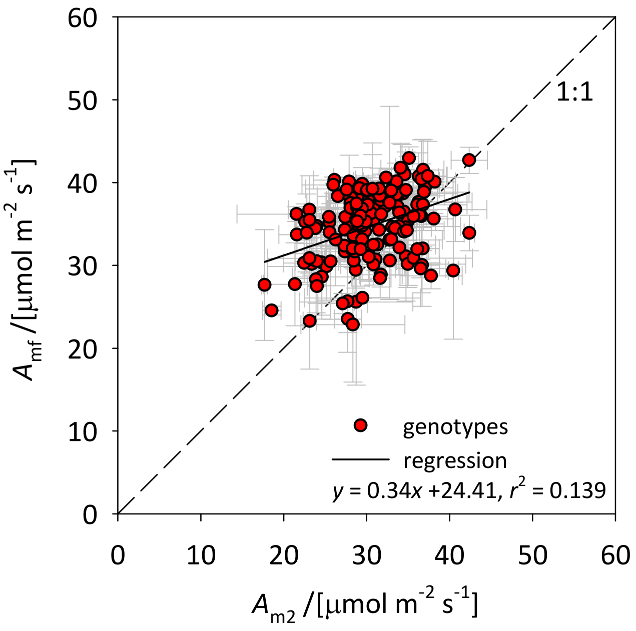

Figure S6. Flag leaf A_m vs second leaf A_m. n = 160 genotypes.

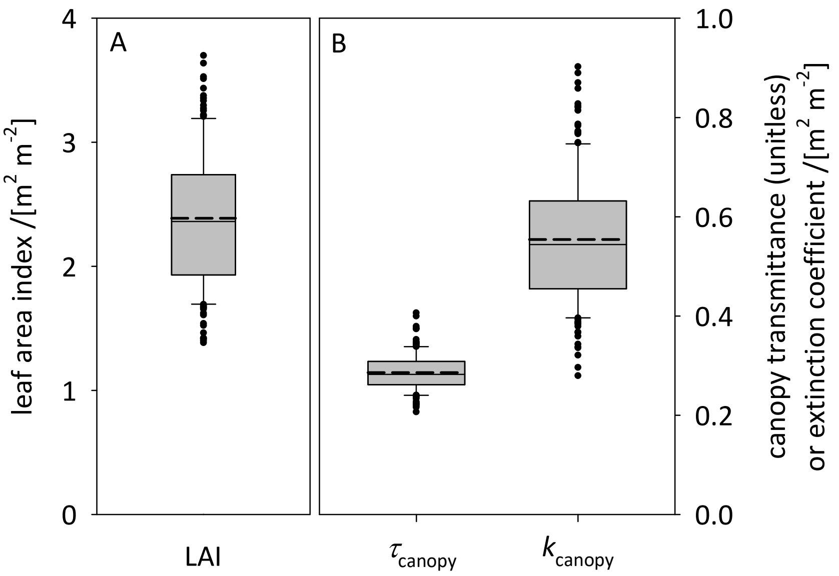

Figure S7. Boxplots illustrating distributions of (A) LAI, and (B) canopy transmittance and effective canopy extinction coefficient across genotypes. Boxes denote the interquartile range (25th to 75th percentile); whiskers denote the 10th and 90th percentiles; closed symbols denote outliers; and solid and dashed lines in the boxes denote medians and means, respectively. *n* = 160 genotypes.

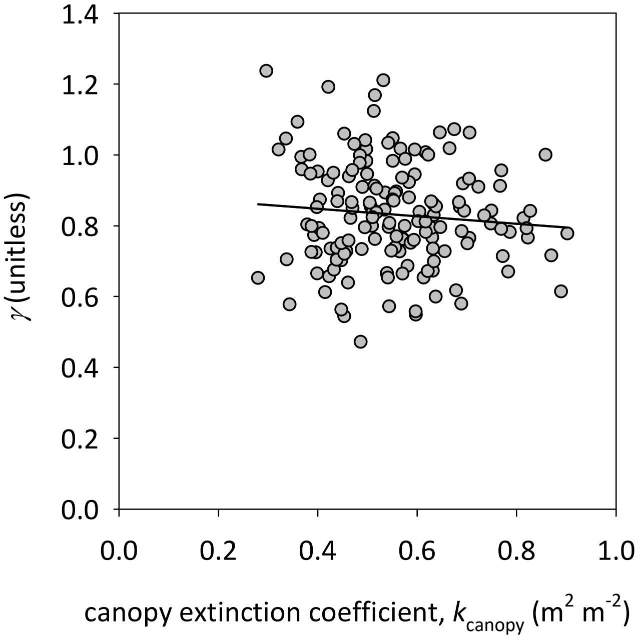

Figure S8. γ was not significantly correlated with canopy structure, as gauged by the effective canopy extinction coefficient, k_{canopy} (y = -0.106x + 0.891, r^2 = 0.003, p > 0.05). n = 160 genotypes.

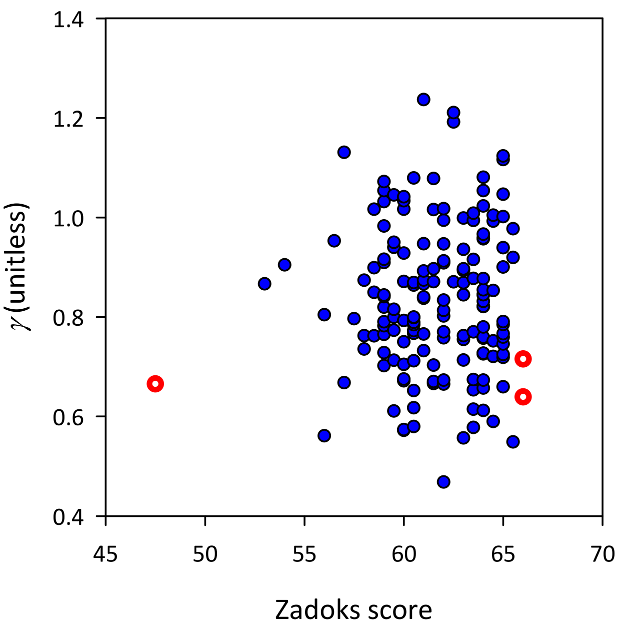

Figure S9. γ was not significantly correlated with phenology (Zadoks score) (regression including all data: p = 0.86, r^2 = 0.0002; regression including the three outliers shown with open red symbols: p = 0.99, r^2 = 0.0000013). n = 160 genotypes.

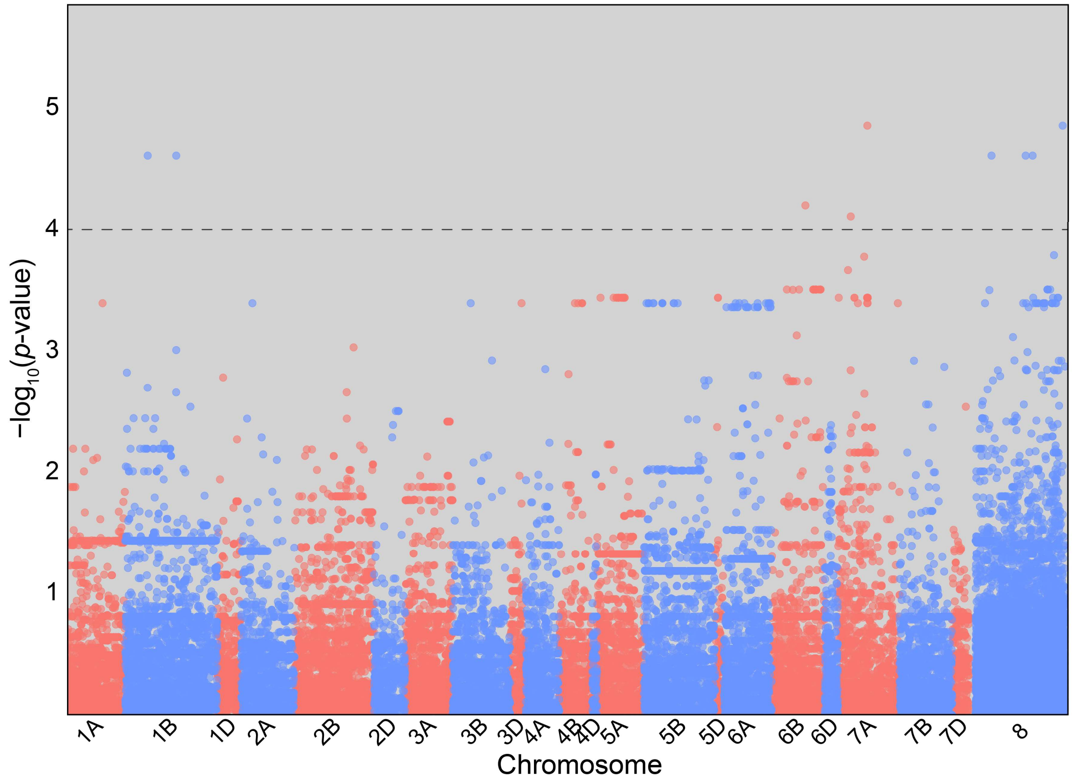

Figure S10. Manhattan plot displaying SNP marker-trait associations identified for A_{m2} across 118 wheat genotypes. Chromosome 8 represents positional unknown SNPs, including those significantly associated with markers 66495_7B, 7806_1B, 47867_4A and 65626_5A in Table 1. The dashed horizontal line represents a significance level of -log10(*p*) = 4, marker/trait associations above this line were deemed significant.

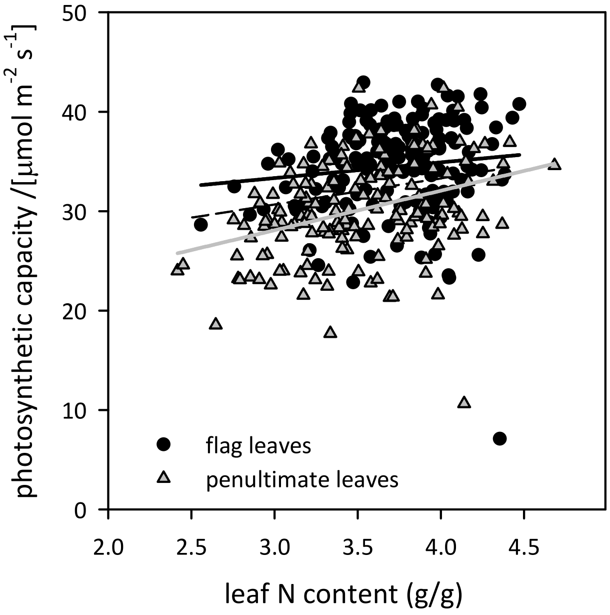

Figure S11. Leaf N content was a poor predictor of photosynthetic capacity. Lines indicate regressions: black line (flag leaves; $y = 1.6x + 28.6$; $r^2 = 0.007$; n.s.), grey line (penultimate leaves; $y = 4.0x + 16.2$; $r^2 = 0.12$; $p < 0.0001$), dotted line (flag and penultimate leaves combined; $y = 2.6x + 22.8$; $r^2 = 0.05$; $p < 0.005$). $n = 160$ genotypes.

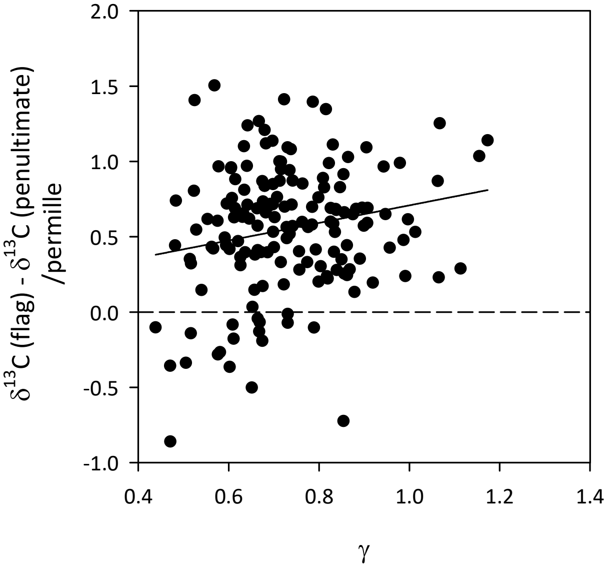

Figure S12. The difference in carbon isotope discrimination ($\delta^{13}C$) between flag and penultimate leaves was larger in magnitude in genotypes with high γ, but the relationship was very weak ($y = 0.584x + 0.125$, $r^2 = 0.033$, $p < 0.05$). n = 160 genotypes.

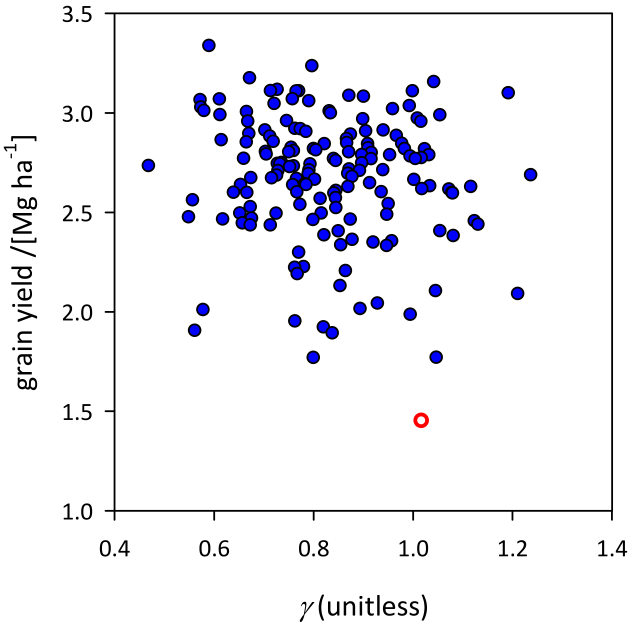

Figure S13. γ was not significantly correlated with grain yield (regression including all data: p = 0.099, r^2 = 0.017; regression including the outlier shown with an open red symbol: p = 0.18, r^2 = 0.011). n = 160 genotypes.
